# Supplementary material for: CD200R deletion promotes a neutrophil niche for Francisella tularensis and increases infectious burden and mortality
Source: Nat Commun. 2019 May 9;10:2121. doi: 10.1038/s41467-019-10156-6 (PMC6509168; doi:10.1038/s41467-019-10156-6)
Supplement: Supplementary file 1 — Supplementary Information [file 41467_2019_10156_MOESM1_ESM.pdf]

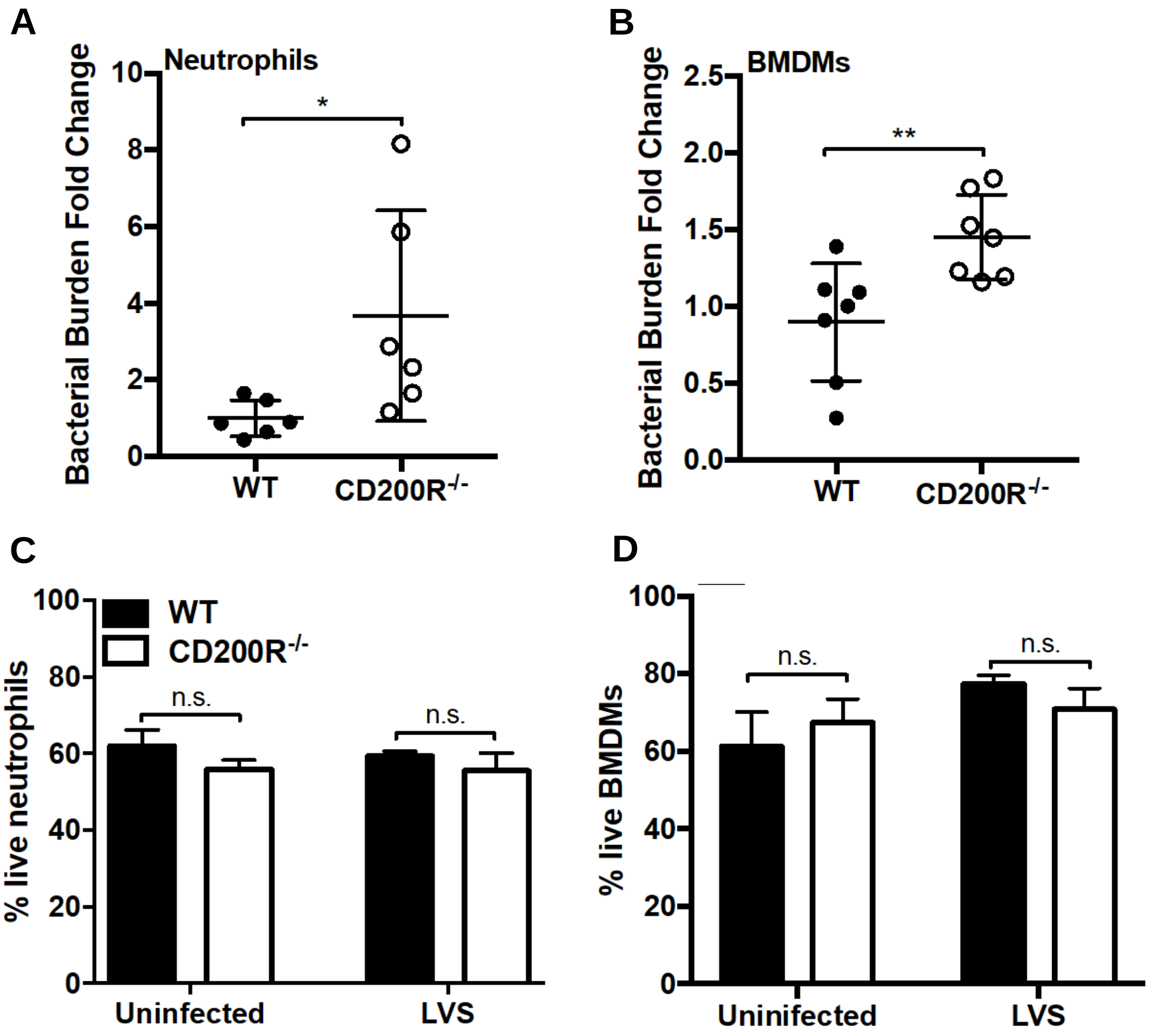


**Supplementary Figure 1. Increased bacterial burden in CD200R$^{-/-}$-derived neutrophils and BMDM despite no difference in cell viability.** Primary BMDM or bone marrow neutrophils were derived from WT or CD200R$^{-/-}$ mice and infected with *F. tularensis* MOI 100. Bacterial burden fold change of CD200R$^{-/-}$-derived neutrophils (A) and BMDM (B) was compared to WT 24 hours post-infection. Cell viability was measured in WT- and CD200R$^{-/-}$-derived neutrophils (C) and BMDM (D) cultured for 24 hours with media alone (uninfected) or *F. tularensis* LVS. Data represent two independent experiments (A-B, n=6-7) or one independent experiment (C-D, n=3) with data shown as mean ± SD (n=6-7). Statistical analysis was performed using unpaired t-tests (A-B) and two-way ANOVA (C-D) (*p<0.05).

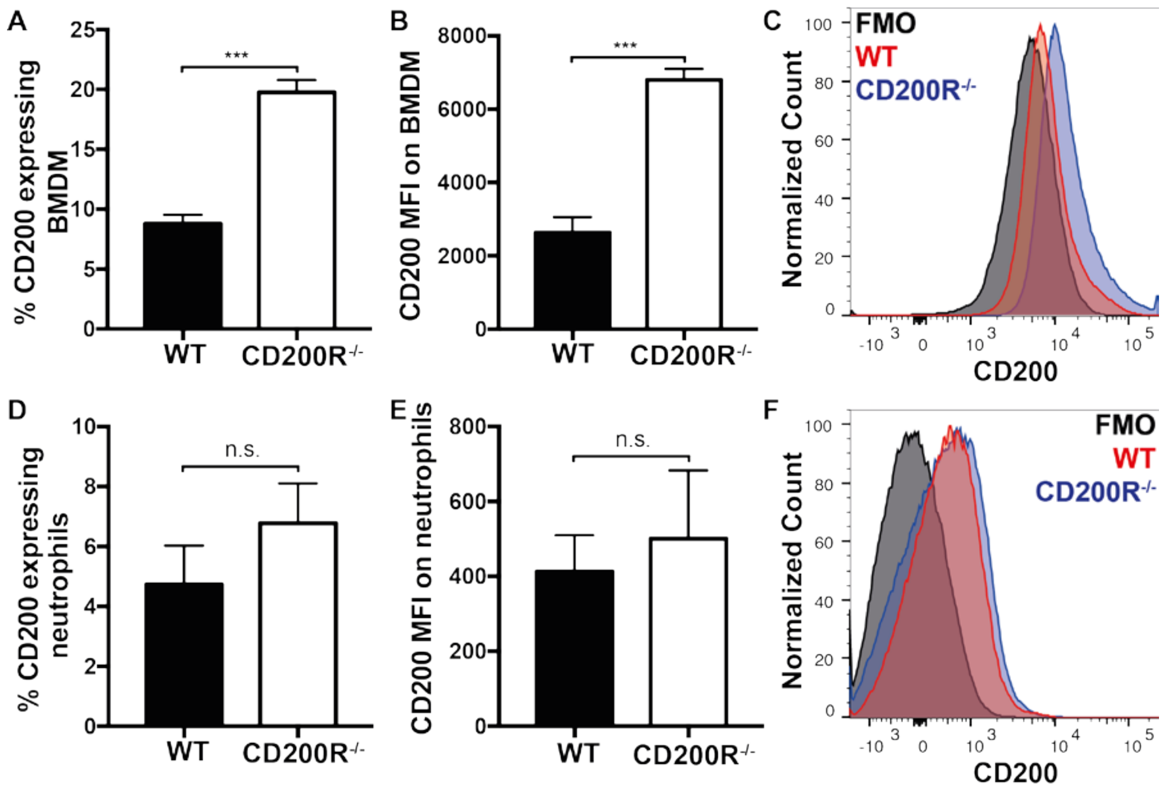


**Supplementary Figure 2. Analysis of CD200 expression on WT and CD200R$^{-/-}$ bone marrow-derived macrophages and neutrophils.** Primary BMDM were derived from WT and CD200R$^{-/-}$ mice and A) CD200 expression and B) CD200 MFI was measured by flow cytometry. C) Representative histogram of CD200 fluorescence intensity in WT (red) or CD200R$^{-/-}$ (blue) BMDM, with control (black). Neutrophils (CD45$^{+}$CD11b$^{+}$Ly6G$^{+}$) were gated within bone marrow from WT and CD200R$^{-/-}$ mice and D) CD200 expression and E) CD200 MFI was measured by flow cytometry. F) Representative histogram of CD200 fluorescence intensity in WT (red) or CD200R$^{-/-}$ (blue) bone marrow neutrophils, with control (black). Data is n=3, and is shown as mean ± SD. Statistical analysis was performed using unpaired t-tests (***p<0.001).

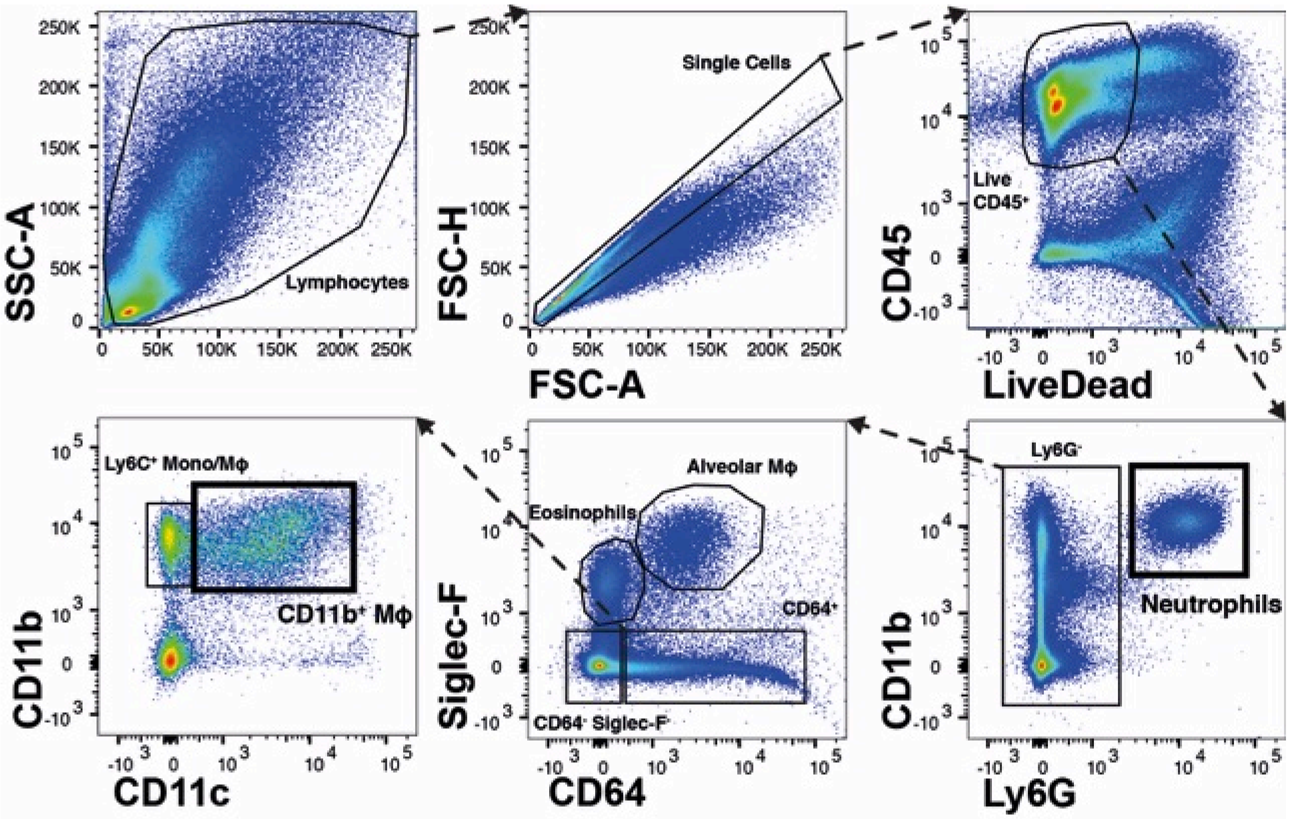


**Supplementary Figure 3. Gating strategy for neutrophils and CD11b+ macrophages in the lung.** Lungs were analysed by flow cytometry, gating strategy on single, live, CD45+ cells. Neutrophils were then identified as Ly6G+CD11b+, while CD11b+ macrophages were further gated from the Ly6G-Siglec-F-CD64+ population.

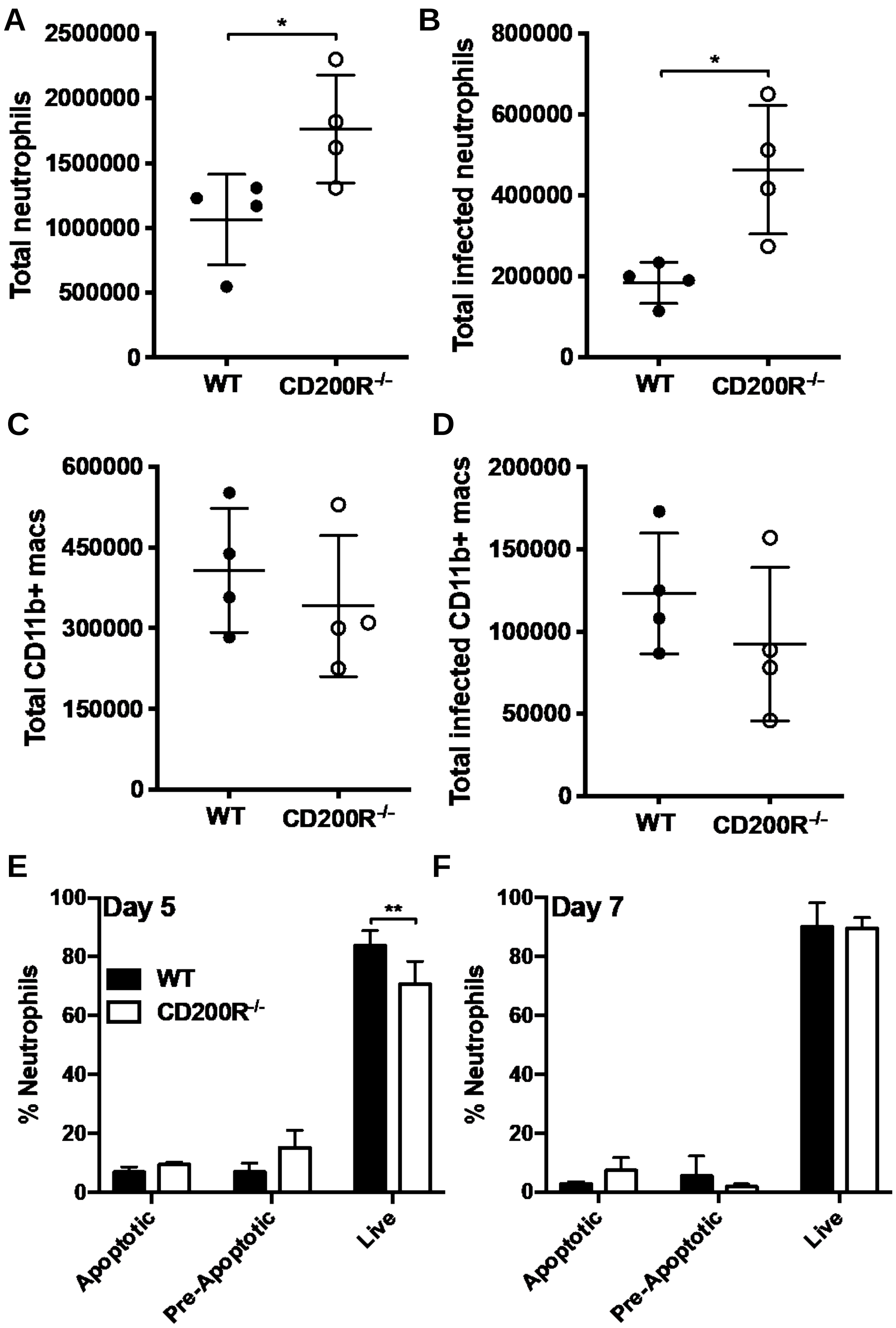


**Supplementary Figure 4. CD200R$^{-/-}$ mice have enhanced numbers of total and infected neutrophils, but not macrophages at day 7 p.i. with *F. tularensis*.** WT and CD200R$^{-/-}$ C57BL/6 mice were infected IN with a challenge dose of 1000 CFU *F. tularensis* LVS. A) Total cell numbers of neutrophils and B) total *F.* tularensis-infected neutrophils in the lung of WT and CD200R$^{-/-}$ mice at day 7 p.i. determined by flow cytometry C) Total cell numbers of CD11b+ macrophages and D) total *F.* tularensis-infected CD11b+ macrophages in the lung of WT and CD200R$^{-/-}$ mice determined by flow cytometry. Data is representative of two independent experiments (n=4) and is shown as mean ± SD. Statistical analysis was performed using unpaired t-test (*p<0.05). Cell death progression in neutrophils (CD11b$^{+}$Ly6G$^{+}$) was monitored at E) day 5 and F) day 7 p.i. Cell death progression was gated accordingly: apoptotic cells (AnnexinV$^{+}$ZombieUV$^{+}$), pre-apoptotic cells (AnnexinV$^{+}$ZombieUV$^{-}$) and live cells (AnnexinV$^{-}$ZombieUV$^{-}$). Data represents one experiment and is shown as mean ± SD (n=4). Statistical analysis was performed using two-way ANOVA (**p<0.01).

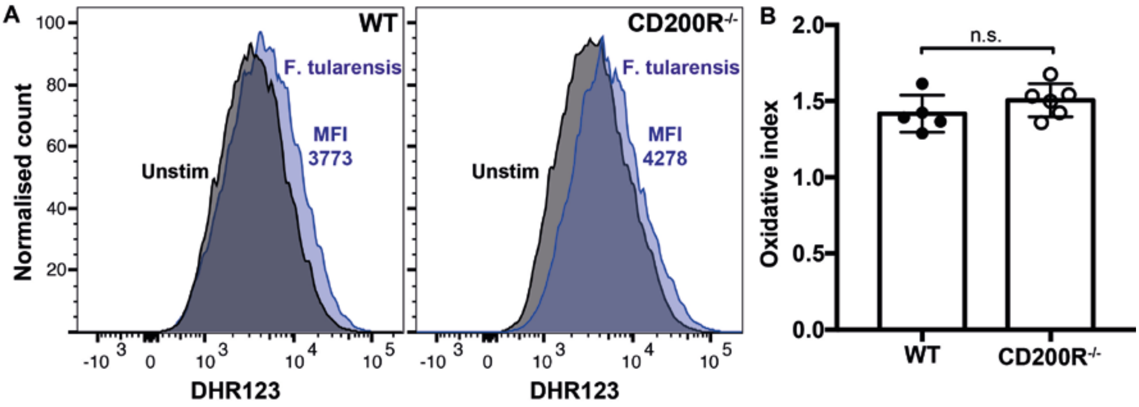


**Supplementary Figure 5. No difference in ROS output between WT and CD200R$^{-/-}$-derived BMDM.** A) Representative histograms of FITC DHR123 expression in unstimulated and *F. tularensis*-infected BMDM at 24 hours p.i. B) Oxidative index was quantified using the following equation: MFI *F. tularensis*-infected/MFI unstimulated. Data represents two independent experiments and is shown as mean ± SD (n=5-6). Statistical analysis was performed using unpaired t-test.
