# Supplementary material for: CD200R deletion promotes a neutrophil niche for Francisella tularensis and increases infectious burden and mortality
Source: Nat Commun. 2019 May 9;10:2121. doi: 10.1038/s41467-019-10156-6 (PMC6509168; doi:10.1038/s41467-019-10156-6)
Supplement: Supplementary file 3 — Reporting Summary [file 41467_2019_10156_MOESM3_ESM.pdf]

nature research

Corresponding author(s): DBPR NCOMMS-18-16922
Last updated by author(s): Apr 8, 2019

Reporting Summary

Nature Research wishes to improve the reproducibility of the work that we publish. This form provides structure for consistency and transparency in reporting. For further information on Nature Research policies, see Authors & Referees and the Editorial Policy Checklist.

Statistics

For all statistical analyses, confirm that the following items are present in the figure legend, table legend, main text, or Methods section.

n/a	Confirmed	
☐	☒	The exact sample size (*n*) for each experimental group/condition, given as a discrete number and unit of measurement
☐	☒	A statement on whether measurements were taken from distinct samples or whether the same sample was measured repeatedly
☐	☒	The statistical test(s) used AND whether they are one- or two-sided<br>*Only common tests should be described solely by name; describe more complex techniques in the Methods section.*
☐	☒	A description of all covariates tested
☐	☒	A description of any assumptions or corrections, such as tests of normality and adjustment for multiple comparisons
☐	☒	A full description of the statistical parameters including central tendency (e.g. means) or other basic estimates (e.g. regression coefficient) AND variation (e.g. standard deviation) or associated estimates of uncertainty (e.g. confidence intervals)
☒	☐	For null hypothesis testing, the test statistic (e.g. *F*, *t*, *r*) with confidence intervals, effect sizes, degrees of freedom and *P* value noted<br>*Give P values as exact values whenever suitable.*
☒	☐	For Bayesian analysis, information on the choice of priors and Markov chain Monte Carlo settings
☒	☐	For hierarchical and complex designs, identification of the appropriate level for tests and full reporting of outcomes
☒	☐	Estimates of effect sizes (e.g. Cohen's *d*, Pearson's *r*), indicating how they were calculated

Our web collection on statistics for biologists contains articles on many of the points above.

Software and code

Policy information about availability of computer code

Data collection	Illumina HiSeq4000 for RNA-seq data colection
Data analysis	Read trimming was carried out with Trimmomatic and quality control was validated with FastQC software (Babraham Bioinformatics). Pseudoalignment was performed with Kallisto to generate transcript abundance estimates. Tximport was applied to create a gene-level count matrix that was further analysed with DESeq2 package to perform differential expression analysis. Finally, an unsupervised gene clustering analysis was applied with pheatmap package using a mean centring scaling.

For manuscripts utilizing custom algorithms or software that are central to the research but not yet described in published literature, software must be made available to editors/reviewers. We strongly encourage code deposition in a community repository (e.g. GitHub). See the Nature Research guidelines for submitting code & software for further information.

Data

Policy information about availability of data

All manuscripts must include a data availability statement. This statement should provide the following information, where applicable:

- Accession codes, unique identifiers, or web links for publicly available datasets
- A list of figures that have associated raw data
- A description of any restrictions on data availability

The datasets generated during and/or analysed during the current study are available from the corresponding author on reasonable request. RNA-seq generated as part of figure 4 has been deposited in GEO Bank (accession number GSE129287) [https://www.ncbi.nlm.nih.gov/geo/query/acc.cgi?acc=GSE129287].

Field-specific reporting

Please select the one below that is the best fit for your research. If you are not sure, read the appropriate sections before making your selection.

☒ Life sciences ☐ Behavioural & social sciences ☐ Ecological, evolutionary & environmental sciences

For a reference copy of the document with all sections, see nature.com/documents/nr-reporting-summary-flat.pdf

Life sciences study design

All studies must disclose on these points even when the disclosure is negative.

Sample size	For mouse experimentation, we planned experimental numbers to be used with Prof. Richard Preziosi, who was lead for biostatistics and an expert on experimental design/power calculations at the University of Manchester. Calculations estimated that, to detect a 20% difference with a ~10% error rate, ~6 animals per condition were required. In many experiments we analysed more animals, as when looking at new parameters we could also analyse parameters (e.g. bacterial burden) that we had previously looked at.
Data exclusions	No data were excluded from the analyses.
Replication	Experiments were repeated with at least two to three biologically independent studies for all results presented in the main manuscript.
Randomization	We did not use randomization to assign animals to experimental groups. Animals were age and sex-matched.
Blinding	Blinding was not necessary in our studies as there were no subjective analyses.

Reporting for specific materials, systems and methods

We require information from authors about some types of materials, experimental systems and methods used in many studies. Here, indicate whether each material, system or method listed is relevant to your study. If you are not sure if a list item applies to your research, read the appropriate section before selecting a response.

Materials & experimental systems

n/a	Involved in the study	
☐	☒	Antibodies
☒	☐	Eukaryotic cell lines
☒	☐	Palaeontology
☐	☒	Animals and other organisms
☒	☐	Human research participants
☒	☐	Clinical data

Methods

n/a	Involved in the study	
☒	☐	ChIP-seq
☐	☒	Flow cytometry
☒	☐	MRI-based neuroimaging

Antibodies

Antibodies used	Descriptions of antibodies used (including concentration, supplier name, clone name and catalog number) can be found in methods section 'Flow cytometry'.
Validation	All flow cytometry antibodies are commercially available for staining mouse samples and validated by the manufacturer.

Animals and other organisms

Policy information about studies involving animals; ARRIVE guidelines recommended for reporting animal research

Laboratory animals	In vivo experiments consisted of 8-10 week old female wild-type C57BL/6 (Charles River, UK) or CD200R-/- mice developed on a C57BL/6 background (as described in 'Mice & in vivo F. tularensis infection' Methods section.
Wild animals	No wild animals were included.
Field-collected samples	No field-collected samples were included.
Ethics oversight	Mice were kept in specific pathogen–free conditions according to institutional and UK Home Office guidelines in the Biological Services Unit at The University of Manchester.

Note that full information on the approval of the study protocol must also be provided in the manuscript.

Flow Cytometry

Plots

Confirm that:

☒ The axis labels state the marker and fluorochrome used (e.g. CD4-FITC).

☒ The axis scales are clearly visible. Include numbers along axes only for bottom left plot of group (a 'group' is an analysis of identical markers).

☒ All plots are contour plots with outliers or pseudocolor plots.

☒ A numerical value for number of cells or percentage (with statistics) is provided.

Methodology

Sample preparation	Sample preparation protocol is describe in Methods section 'Digestion of lung tissue' and 'Flow cytometry'.
Instrument	LSR Fortessa and FACS Canto II (BD Biosciences)
Software	Flow cytometry data were collected via Diva (BD Biosciences) and analyzed by FlowJo V10 (TreeStar).
Cell population abundance	No cell sorting was included.
Gating strategy	The gating strategy for in vivo studies is described in Supplementary Figure 3

☒ Tick this box to confirm that a figure exemplifying the gating strategy is provided in the Supplementary Information.
