# Supplementary material for: CD200R deletion promotes a neutrophil niche for Francisella tularensis and increases infectious burden and mortality
Source: Nat Commun. 2019 May 9;10:2121. doi: 10.1038/s41467-019-10156-6 (PMC6509168; doi:10.1038/s41467-019-10156-6)
Supplement: Supplementary file 4 — Source Data [file 41467_2019_10156_MOESM4_ESM.pdf]

Figure 1B

	2 hours	24 hours
WT	18.1	60.5
	17.4	58.1
	18.4	50.7
	17.3	49.2
	18.9	54.6
$CD200R^{-/-}$	21.7	67.3
	21.8	64.9
	24.1	69.3
	31.2	69.8
	28.9	62.1

Figure 1D

	4 hours	24 hours
WT	1.98	43.6
	3.01	36.9
	3.71	31.8
	3.55	37.6
	10.9	39.6
$CD200R^{-/-}$	4.88	53.4
	4.43	43.9
	3.16	47.9
	14.5	48.5
	11.8	50
	11.7	42.1

Figure 2A

	Survive Day 7	Dead Day 5	Dead Day 6	Dead Day 7
**WT**	28	0	0	1
**CD200R$^{-/-}$**	21	3	3	5

Figure 2B

**WT**					
**Day 1**	**Day 3**	**Day 5**	**Day 7**		
117000	7200000	9140000	12100000	14400000	9350000
200000	4670000	7330000	12500000	12500000	11100000
133000	6110000	17700000	2950000	841000	14800000
162000	7670000	9360000	5900000	8000000	12200000
122000	27500000	10000000	14800000	5600000	9290000
132000	22800000	9900000	12200000	12500000	14400000
	34100000	12200000	9290000	10900000	

**CD200R$^{-/-}$**					
**Day 1**	**Day 3**	**Day 5**	**Day 7**		
167000	2000000	1420000	11900000	507000	293000000
233000	14200000	4330000	11800000	733000	158000000
133000	12400000	12800000	19400000	10400000	
150000	14900000	9240000	20100000	4210000	
80700	32400000	13300000	19600000	38100000	
45900	46700000	19500000	#########	46100000	
202000	15900000	47000000	#########	19800000	
144000		38300000	3290000	30500000	

Figure 2C

**WT**				**CD200R-/-**			
**Day 1**	**Day 3**	**Day 5**	**Day 7**	**Day 1**	**Day 3**	**Day 5**	**Day 7**
0.48	0.43	0.63	1.06	0.27	0.28	0.67	1.12
0.36	0.46	0.38	0.73	0.22	0.45	0.57	1.27
0.37	0.38	0.63	0.92	0.30	0.39	0.79	1.23
0.36	0.47	0.77	1.24	0.37	0.35	0.63	1.17
0.30	0.44	0.66	0.69	0.53	0.51	0.52	1.38
0.30	0.42	0.83	0.60	0.56	0.57	0.56	0.54
0.31	0.46	1.03	0.96	0.34	0.42	0.68	0.90
0.24			0.65	0.38		0.57	0.99
							1.05

Figure 2D

	WT			
**Day 1**	24.6	10.1	6.89	5.88
**Day 3**	22.5	25.4	24.7	
**Day 5**	25.6	31.9	25.5	26.2
**Day 7**	24.1	24.3	31.3	25.9
	$CD200R^{-/-}$			
**Day 1**	11.2	7.31	8.5	7.74
**Day 3**	26.6	23.5	21.3	
**Day 5**	44.2	37.7	37.3	36.6
**Day 7**	35.1	34	34.1	

Figure 2E

	WT			
**Day 1**	4.25	6.21	5.36	6.27
**Day 3**	13.4	14.9		
**Day 5**	20.5	24.5	26.2	27.7
**Day 7**	9.94	8.37	13.2	7.22
	$CD200R^{-/-}$			
**Day 1**	4.44	8.270001	8.31	6.78
**Day 3**	23.6	23.5	15.8	
**Day 5**	26.6	37.1	40.8	37.7
**Day 7**	17.8	23.5	19	

Figure 2F

	WT			
**Day 1**	9.92	8.26	10.9	9.83
**Day 3**	10.2	6.39		
**Day 5**	36	24.2	24.4	25.2
**Day 7**	37.2	38.1	33.5	
	$CD200R^{-/-}$			
**Day 1**	8.07	6.53	7.03	8.75
**Day 3**	16.4	16.7	11.8	
**Day 5**	18.7	19	16.6	23.6
**Day 7**	30.2	28.1	33.2	

Figure 2G

	WT			
**Day 1**	36	25.5	29.5	35.1
**Day 3**	15.6	10.6		
**Day 5**	17.3	18.3	17.1	19.6
**Day 7**	12.6	14.8	19.3	
	**CD200R$^{-/-}$**			
**Day 1**	31.1	26	24.2	25.8
**Day 3**	14.9	13.2	21	
**Day 5**	16.8	19	22.1	21
**Day 7**	21.3	17.1	19.8	16.4

Figure 2H

hIgG	CD200-Fc
183000000	96300000
152000000	76700000
156000000	123000000
203000000	103000000

Figure 2I

hIgG	CD200-Fc
1.05	0.17
0.88	0.44
0.90	0.71
1.17	0.59
1.84	0.24
0.59	0.33
0.64	0.41
0.36	0.39
1.57	

Figure 2J

hIgG	CD200-Fc
24.3	17.4
25.1	19.2
22	19.4
22.1	21.3
19.3	

Figure 3B

WT		CD200R-/-	
IgG2a	αLy6G	IgG2a	αLy6G
9.48	1.78	24.6	2.45
17.1	1.39	25.7	3.63
13.2	0.9	26.2	3.38
13.9	1.6	21.9	1.83
17.3	0.91	22.2	1.85
17.1	4.92	20.2	4.3
17.2	3.79	19.6	2.39
14.6	2.75	15.7	2.17
12.1	1.92	19	2.36
18.2	2.31	16.5	3.02
11.3	0.96	16.6	2.17
14.4	0.88	18.1	2.62
12.2	1.46	19.1	3.65
13.4	1.6	14.4	2.49
19	2.82		1.36

Figure 3C

WT		CD200R-/-	
IgG2a	αLy6G	IgG2a	αLy6G
2333333.33	427000	5850000	6570000
762000	5470000	6440000	3040000
6930000	3200000	16100000	11700000
2760000	8890000	8000000	10000000
2600000	7520000	6200000	8930000
7830000	8180000	15800000	6080000
3780000	12400000	28300000	15200000
18100000	3280000	18400000	12100000
9190000	2260000	21900000	7800000
5920000	1520000	45600000	10800000
12000000	17100000	80000000	5760000
		53800000	6740000
		9390000	7700000
		11200000	15500000
		4190000	9580000

Figure 4A

Ensembl ID	Sample 1 - WT1	Sample 2 - WT2	Sample 3 - WT3	Sample 4 - KO1	Sample 5 - KO2	Sample 6 - KO3
ENSMUSG00000005413 - Hmox1	-0.893	-1.045	-1.036	0.955	1.042	0.978
ENSMUSG00000022982 - Sod1	-0.579	-0.438	-0.458	0.503	0.644	0.327
ENSMUSG00000025289 - Prdx4	-0.192	-0.273	-0.215	0.202	0.277	0.201
ENSMUSG00000050708 - Ftl1	-0.441	-0.542	-0.425	0.364	0.647	0.396
ENSMUSG00000037710 - Cisd1	-0.298	-0.184	-0.184	0.322	0.259	0.085
ENSMUSG00000028691 - Prdx1	-0.469	-0.543	-0.483	0.658	0.453	0.385
ENSMUSG00000020250 - Txnrd1	-0.181	-0.230	-0.252	0.204	0.235	0.224

Figure 4C

WT	$CD200R^{-/-}$
74.91	32.09
47.59	45.01
43.50	27.82
18.77	16.81
20.61	20.59
19.77	18.45
120.81	64.84
66.23	47.12
110.56	33.51
59.40	28.33
54.51	23.75
47.91	37.67

Figure 4E

WT	$CD200R^{-/-}$
1.94	1.52
1.88	1.38
1.79	1.35
2.51	1.73
3.81	2.05
2.56	1.67

Figure S1A

WT	CD200R$^{-/-}$
0.442	1.654
0.905	1.169
1.654	2.332
0.643	5.859
0.874	8.162
1.483	2.879

Figure S1B

WT	CD200R$^{-/-}$
0.908	1.193
1.091	1.229
1.000	1.445
1.107	1.832
1.389	1.157
0.504	1.526
0.271	1.771

Figure S1C

		**WT**	
**Uninfected**	63.5	65.3	57.4
**LVS**	58.9	60.8	58.3
		**CD200R$^{-/-}$**	
**Uninfected**	55.3	58.5	53.8
**LVS**	60.4	54.9	51.4

Figure S1D

		**WT**	
**Uninifected**	51	64.7	67.8
**LVS**	75.8	76.6	79.8
		**CD200R$^{-/-}$**	
**Uninfected**	66.2	62	74
**LVS**	75.6	64.9	71.8

Figure S2A

WT	CD200R-/-
8.05	18.7
8.8	20.7
9.52	19.9

Figure S2B

WT	$CD200R^{-/-}$
2290	6452
2528	7060
3100	6872

Figure S2D

WT	$CD200R^{-/-}$
3.24	5.26
5.63	7.77
5.33	7.3

Figure S2E

WT	$CD200R^{-/-}$
302	345
480	454
457	702

Figure S4A

WT	$CD200R^{-/-}$
1230000	1820000
1310000	1620000
1170000	2300000
547000	1310000

Figure S4B

WT	$CD200R^{-/-}$
190000	512000
234000	417000
200000	651000
114000	274000

Figure S4C

WT	$CD200R^{-/-}$
357000	310000
438000	225000
551000	529000
283000	300000

Figure S4D

WT	$CD200R^{-/-}$
108000	88700
125000	45900
173000	157000
86700	78000

Figure S4E

	WT			
**Apoptotic**	4.6	7.35	7.08	8.61
**Pre-Apoptoti**	4.47	4.8	7.93	10.7
**Live**	89.5	85.8	82.2	77.6
	$CD200R^{-/-}$			
**Apoptotic**	9.07	9.97	10.15	8.71
**Pre-Apoptoti**	7.44	17	21.4	15
**Live**	80.8	68	62.6	71.9

Figure S4F

	WT			
**Apoptotic**	2.47	2.64	3.77	2.12
**Pre-Apoptoti**	1.43	1.73	2.95	15.6
**Live**	95.5	94.9	92.2	78.2
	$CD200R^{-/-}$			
**Apoptotic**	3.14	6.66	13.47	6.15
**Pre-Apoptoti**	3.1	1.45	1.43	1.48
**Live**	91.4	91.2	84	91.4

Figure S5B

WT	$CD200R^{-/-}$
1.365	1.532
1.393	1.425
1.615	1.360
1.424	1.499
1.289	1.676
	1.543
